# Acknowledgment of Reviewers

**DOI:** 10.1093/geroni/igag005

**Published:** 2026-02-03

Scientific progress depends on the generosity of reviewers who assist editors by sharing their time and expertise in the peer review process. Michelle Putnam, PhD, MGS, FGSA, Editor-in-Chief of *Innovation in Aging*, wishes to thank the following individuals for their assistance in reviewing manuscripts during 2025.

Abaei, Elnaz

Abbott, Katherine

Abrahamson, Kathleen

Adamek, Margaret

Adams, Kathryn

Aflatoony, Leila

Ågren, Axel

Agudelo, Marcela

Ai, Wensi

Akaida, Shoma

Albert, Steven

Aldwin, Carolyn

AlHasan, Dana

Alhiti, Hazim

Ali, Talha

Alkan, Ömer

Allen, Julie

Almroth, Melody

AlZahmi, Amal

Amano, Takashi

Amoako, Emmanuel

Amoin, Cyril

Anderson, Keith

Arora, Kanika

Aula, Inkeri

Avila-Rieger, Justina

Ayalon, Liat

Ayton, Darshini

Badache, Andreea

Bae, Youngsook

Baek, Jihye

Bai, Dorothy

Baik, Dawon

Baik, Sol

Bankole, Azziza

Bao, Huanyu

Barber, Sarah

Barmon, Christina

Batista, Sandro

Baumann, Steven

Beach, Scott

Beauchamp, Mark

Beebe, Silas

Bell, Sue Anne

Beltran, Susanny

Berbon, Caroline

Berkowsky, Ronald

Bethea, Traci

Beydoun, May

Bhaumik, Deepon

Bhiwoo, Yovesh

Bhowmick, Pallabi

Bielsten, Therese

Blotenberg, Iris

Boa Sorte Silva, Narlon

Boer, Dennis

Boffy-Ramirez, Ernest

Boot, Walter

Boron, Julie

Boyd, Larissa

Bozzato, Matteo

Brent, Robert

Bristol, Alycia

Brody, Abraham

Brooks, Amber

Brooks, Deborah

Broome, Emma

Brothers, Allyson

Buczak-Stec, Elzbieta

Bünning, Mareike

Burgdorf, Julia

Burnes, David

Button, Mark

Cabrero Castro,

José Eduardo

Cacchione, Pamela

Cahill, Kevin

Cai, Wenjie

Calvo, Esteban

Camicioli, Richard

Campbell, Kenna

Cao, Yuan

Chan, Andrew

Chan, Athena

Chandola, Tarani

Chang, Pei-Fen

Chang, Wei Hung

Chang, Xiaomin

Chantarat, Tongtan

Chasteen, Alison

Chatterjee, Sudeshna

Chattopadhyay, Debaleena

Chau, Diane

Chen, Chuqian

Chen, Hsi-Chung

Chen, Jia

Chen, Laura

Chen, Liang-Kung

Chen, Limei

Chen, Li-Mei

Chen, Shenglan

Chen, Yen-Chin

Chen, Yu-Chih

Chen, Zhuo

Cheng, Greta Jianjia

Chewning, Betty

Chiu, Chi-Tsun

Cho, Hannah

Cho, Jinmyoung

Cho, Soyeon

Choi, Kyung Won

Choi, Moon

Choi, Namkee

Choi, Sunha

Choy, Jacky

Chrisman, Theresa

Chung, Jane

Cichy, Kelly

Cohen, Trevor

Csete, Joanne

Curl, Angela

Czaja, Sara

Daijo, Shiratsuchi

Dang, Truc Ngoc Hoang

Dasanayake, Thakshila

Dassel, Kara Bottiggi

Davey, Adam

Davitt, Joan

De Lima, Bryanna

Degenholtz, Howard

Dhakal, Usha

Diederich, Freya

Dobrota, Sylvie

Dovie, Delali A.

Dupuis, Kate

Eberly, Lori

Ebner-Priemer, Ulrich

Egbujie, Bonaventure

Elisha, Victor

Elsorady, Khalid

Eppler, Raphael

Erickson, Alexander J.

Essien, Ime

Fabbre, Vanessa

Fahsold, Anne

Faieta, Julie

Faivre, Pierre

Fan, Erica

Fang, Fang

Fariman, Soroush

Farsijani, Samaneh

Feng, Qiushi

Fennell, Gillian

Fernández-Sánchez, Rosa

Fingerman, Karen

Finkel, Deborah

Flatt, Jason

Folbre, Nancy

Fortinsky, Richard

France, Charlene

French, Rachel

Fruhauf, Christine

Fu, Yuanyuan

Fung, Helene

Gallant, Natasha

Gao, Siyu

Garcia Morales, Emmanuel

Gebauer, Sarah

George, Grace

Gerber, Garland

Germain, Cassandra

Gilligan, Megan

Gimm, Gilbert

Giné, Florencia

Glicksman, Allen

Glynn, Nancy

Goins, Ruth

Goodwin, Ashley

Grainger, Lindsay

Greenfield, Emily

Grinshteyn, Erin

Grischott, Thomas

Guo, Xingzhi

Guo, Yu

Haghighi, Paniz

Halpin, Sean

Halvorsen, Cal

Hamler, Tyrone

Hamm, Jeremy

Hammersmith, Anna

Hanes, Douglas

Harel, Dovrat

Harrington, Erin

Harrington, Rosamund

Harris, Carl

Harrison, Jordan

Harrison, Tracie

Hartmann, Patrick

Hasegawa, Yoko

Hass, Zachary

Hawk, Mary

Helzner, Elizabeth

Hertzog, Christopher

Hill, Nikki

Hoell, Andreas

Hoffman, Rashelle

Hollis, Linda

Hong, Y. Alicia

Hsieh, MinJia

Hsu, Hui-Chuan

Hsu, Yen-Hsuan

Hu, Bo

Hu, Jierong

Hua, Cassandra

Huang, Tim

Huang, Xinru

Huang, Yan

Huang, Yaqi

Huang, Yingyan

Hunter, Mary Ann

Iaboni, Andrea

Idler, Ellen

Inoue, Megumi

Jadavji, Nafisa

Jain, Jennifer

Janevic, Mary

Jang, Soeun

Jansen, Carl-Philipp

Jao, Ying-Ling

Jayakody, Dona

Jiang, Nan

Jiang, Yanping

Johansson, Karin

Jones, Janelle

Joo, Jin Hui

Joshi, Herat

Jung, Eun Hwa

Kanning, Martina

Kaplan, Daniel

Karlin, Nancy

Katigbak, Carina

Keisari, Shoshi

Kelly, Audrey

Kennedy, Katherine

Kheirbek, Raya

Kim, Aeri

Kim, Giyeon

Kim, Jinho

Kim, Jong In

Kim, Jung Ki

Kim, Juyeon

Kim, Kyeongmo

Kim, Seoyoun

Kim, Taekyung

Kim, Yeonwoo

Kim-Knauss, Yaeji

Klepacz, Laura

Kono, Ayumi

Kulkarni, Snehal

Kwak, Jung

Kwan, Gerald S.Y.

Lach, Helen

Laffon, Blanca

Lai, Chih-Cheng

Lai, Claudia

Lai, Jun S

Lai, Yi Feng

Latham-Mintus, Kenzie

Lau, Wallis

Lee, Min-Ah

Lee, Othelia

Lee, Yeonjung

Lee, Yookyong

Leggett, Amanda

Lewis, Anna

Lewis, Jordan

Li, Guanzhi

Li, Handong

Li, Hang

Li, Jing

Li, Jiyue

Li, Kevin

Li, Tianyuan

Li, Wenjie

Li, Xiaxia

Li, Yichao

Liang, Jiaming

Lichtenberg, Peter

Lim, Justin

Lin, Pei-Chen

Lin, Sapphire

Litzelman, Kristin

Liu, Darren

Liu, Hui

Liu, Jinyu

Liu, Ruotong

Liu, Siyi

Liu, Xinran

Liu, Yang

Liu, Yiran

Liu, Yujun

Liu, Zhihan

Lokuge, Sachithra

Long, Chengxu

López-Anuarbe, Mónika

Lopez-Garcia, Esther

López-Ortega, Mariana

Lou, Yifan

Lu, Nan

Lu, Peiyi

Luo, Yan

Lyons, Karen

Ma, Lina

Magid, Kate

Maina, Lucy

Malhotra, Rahul

Manalel, Jasmine

Manly, Jennifer

Marques, Mario

Marsh, Zyrene

Martí-Danés, Aleix

Martinez, Iveris

Martire, Lynn M.

Maslow, Katie

Mattos, Meghan

Matz-Costa, Christina

McConnell, Eleanor

McLaughlin, Anne

Mekary, Said

Meng, Hongdao

Menne, Heather

Meyer, Kylie

Michaels, Jacqueline

Miller, Katherine

Mills, Whitney

Miranda, Sandra

Mirza, Raza

Mitchell, Kimberly

Mitchell, Lauren

Mitsutake, Seigo

Miyawaki, Christina

Montepare, Joann

Morris, Earl

Mu, Christina X.

Mudrazija, Stipica

Müller, Anna C.

Murphy, Blake

Myrczik, Janina

Naseh, Mitra

Nelson, Katie

Nemmers, Natasha

Ness, Roberta

Neumann, Lycia

Nguyen, Christopher

Nguyen, Duy

Nguyen, Tran

Nidadavolu, Lolita

Nielsen, Karen

Niznik, Joshua

Obradovic, Natasha

O’Brien, Erica

O’Brien, Marita

O’Grady, Megan

Oh, Oonjee

Oh, Patricia

Oliva, Nancy

Olson, Jenny L.

Ong, Chong Yau

Orlofsky, Sarit

Ortiz Jiménez, Xóchitl

Ory, Marcia

Oswald, Austin

Page, Jeni

Park, Jihyun

Park, Sung

Pascucci, Justin

Patterson, Sarah

Peeters, Geeske

Peng, Tao-Chun

Perepezko, Kate

Pérez-Zepeda, Mario Ulises

Perone, Angela

Peterson, Colleen

Petervari, Erika

Phinney, Alison

Pillemer, Karl

Politi, Julieta

Pope, Natalie

Powers, Sara

Pradhan, Kalandi

Qi, Xiang

Qin, Weidi

Raffin, Jeremy

Resnick, Barbara

Reyes, Jimmy

Rhodes, Annie

Ribeiro, Oscar

Rice, Paige

Ritter, Ashley

Roberto, Karen

Robison, Julie

Romain-Harrott, Melissa

Ross, William

Ruiz, Erik

Russell, David

Ryan, Olivia

Sadarangani, Tina

Sakuma, Misato

Salihu, Ejura Yetunde

Samanta, Tannistha

Sands, Laura

Sanford, Jon

Sanzenbacher, Geoffrey

Savundranayagam, Marie

Schafer, Markus

Schöne, Daniel

Schuster, Amy

Schut, Rebecca

Scrofani, Stephan

Seddon, Johanna

Seo, Jeongone

Shannon, Kay

Shen, Huei-Wern

Shields, Chris

Shin, Hyung Eun

Shuman, Valerie

Siedlecki, Karen

Siette, Joyce

Singnoy, Chatkamon

Skinner, Jared

Skinner, Robert

Snow, Kimberly

Solomon, Diane

Son, Jongsang

Song, Qian

Soske, Jon

Sou, Ka Lon

Stanley, Jennifer

Stark, Susan

Steffen, Ann

Stites, Shana

Stock, Matt

Stokes, Jeffrey

Su, Tai-Te

Suanet, Bianca

Sun, Fei

Sun, Yi

Sung, Pildoo

Szanton, Sarah

Tabloski, Patricia

Taha, Hash brown

Tanaka, Hiroyuki

Tang, Haoxian

Tang, Shangfeng

Tappen, Ruth

Teaster, Pamela

Toh, Wei Xing

Topping, Michael

Toto, Pamela

Toyoshima, Aya

Tucker, Gretchen

Tulloch, Kristen

Turcotte, Samuel

Turner, Shelbie

Tzeng, Huey-Ming Tzeng

Urbanski, Dana

Varilek, Brandon

Venditti, Elizabeth

Vickerstaff, Sarah

Waldhauser, Katrina

Wang, Dahua

Wang, Hanqian

Wang, Kaipeng

Wang, Kaixian

Wang, Sijiu

Wang, Wen

Wang, Wenjin

Wang, Yinhua

Wang, Zih-Ling

Wang, Ziyue

Wangmo, Tenzin

Warner-Maron, Ilene

Welburn, Sharon

Weldrick, Rachel

Whetung, Cliff

Wilkinson, Lindsay

Wilkinson, Renae

Williams, Joy

Wilson, Nikki-Anne

Windsor, Tim

Wollesen, Bettina

Woo, Jean

Wu, Bei

Wu, Jiajun

Wu, Jingyi

Wu, Yijin

Wynn, Matthew

Xiang, Xiaoling

Xing, Cai

Xu, Dongjuan

Xu, Hanzhang

Xu, Shu

Yan, Binjian

Yan, Huang-Ting

Yan, Lijing L.

Yan, Mengzhao

Yang, Deng-Chi

Yang, Jie

Yang, Li-Zhuang

Yang, Tse-Chuan

Yang, Yijian

Yang, Yong

Yao, Yao

Yap, Mei Chen

Ye, Minzhi

Young, Peter

Yow, W. Quin

Yu, Jiao

Yu, Xiru

Yuan, Jiameng

Zai, Xianhua

Zajacova, Anna

Zanwar, Preeti

Zeng, Ziwei

Zhan, Hao

Zhang, Jiayu

Zhang, Jinbao

Zhang, Shenghao

Zhang, Zhang

Zhang, Zhenmei

Zhao, Ivy Y

Zheng, Anqing

Zhong, Chuwen

Zhou, Jia-Jia

Zhou, Junhong

Zhou, Wenjian

Zhou, Zexi

Zhou, Zi

Zhuolin, Chen

Zou, Qi

Zurlo, Karen

